# Determination and Ecological Risk Assessment of Quinolone Antibiotics in Drinking and Environmental Waters Using Fully Automated Disk-Based SPE Coupled with UPLC–MS/MS

**DOI:** 10.3390/molecules29194611

**Published:** 2024-09-28

**Authors:** Hongmei Hu, Xingyu Da, Zhenhua Li, Tiejun Li, Xiaoning Zhang, Tianbin Bian, Yanjian Jin, Kaida Xu, Yuanming Guo

**Affiliations:** 1 Key Laboratory of Sustainable Utilization of Technology Research for Fisheries Resources of Zhejiang Province, Zhejiang Marine Fisheries Research Institute, Zhoushan 316021, China; huhm@zju.edu.cn (H.H.);; 2 State Key Laboratory of Resource Insects, College of Sericulture, Textile and Biomass Sciences, Southwest University, Chongqing 400715, China; 3 Hangzhou Center for Disease Control and Prevention, Hangzhou 310021, China; 4 Zhejiang Marine Ecology and Environment Monitoring Center, Zhoushan 316021, China

**Keywords:** quinolone antibiotics, automated disk-based solid-phase extraction, ultra-performance liquid chromatography–tandem mass spectrometry, ecological risk, water

## Abstract

Quinolone antibiotics (QNs) contamination in the aquatic environment is a global public health issue considering their resistance and mobility. In this study, a simple, efficient, and sensitive method was developed for the accurate quantification of fifteen QNs in water using automated disk-based solid-phase extraction (SPE) coupled with ultra-performance liquid chromatography–tandem mass spectrometry (UPLC–MS/MS). By utilizing a 3M SDB-XC disk to enrich QNs from a 1000 mL water sample, the detection limits were improved to 0.008–0.055 ng/L due to the satisfactory enrichment factors of 897−1136, but only requiring about 60 min per six samples. The linearity of the method ranged from 0.05 to 100 μg/L for the 15 QNs, with correlation coefficients of 0.9992–0.9999, and the recoveries were in the range of 81–114%, with relative standard deviations of 0.2–13.3% (n = 6). The developed method was applicable for the quantification of trace QNs at low ng/L levels in drinking and environmental waters. The results showed that no QNs were detected in tap water, while three and four QNs were detected in the river water of Zhoushan and the seawater of Daiquyang and Yueqing Bay, East China, respectively, with a total concentration of 1.600–8.511 ng/L and 1.651–16.421 ng/L, respectively. Among the detected QNs, ofloxacin (OFL) was the predominant compound in river water, while enrofloxacin (ENR) was predominant in seawater. The risk quotient (RQ) results revealed that QNs posed a low risk to crustaceans and fish, but a low-to-medium risk to algae, and OFL presented the main ecological risk factor in river water, while ENR and CIP in seawater. Overall, the proposed automated disk-based SPE–UPLC–MS/MS method is highly efficient and sensitive, making it suitable for routine analysis of QNs in drinking and environmental waters.

## 1. Introduction

As one of the greatest medical discoveries of the 20th century, antibiotics have played an indelible role in treating diseases, improving health, increasing life expectancy, and promoting plant and animal growth [1]. However, the inappropriate use and overuse of antibiotics, along with the resulting global spread of antimicrobial resistance (AMR), have emerged as one of the greatest public health threats in the 21st century [2,3], especially in low- and middle-income countries (LMICs) [4]. At present, there are more than 9000 natural and synthetic antibiotics in the world [5]. Among them, quinolone antibiotics (QNs) are one of the most widely used synthetic antibiotics, with a 4-quinolyl structure, in both human and veterinary medicine, as well as animal growth promoters [6]. Nevertheless, up to 70% of QNs will eventually be excreted into the environment in the form of raw materials or by-products due to their limited absorption or metabolism in humans or animals after administration [7]. They have been detected in surface waters (e.g., rivers, lakes, and offshore waters) [8,9], groundwater [9,10], and even drinking water [11,12] all over the world, with concentrations in the order of ng/L–μg/L, posing hazards to sensitive ecosystems and human health. In this case, it is of great significance to accurately monitor QNs in the water environment and assess their potential health and ecological risks.

Currently, various methods based on microbial/immunoassay, chromatography/mass spectrometry and optical/electrochemical sensing have been established for QNs detection, providing varying levels of sensitivity, selectivity, and applicability [13]. Among these technologies, the chromatography/mass spectrometry methods, including capillary electrophoresis (CE) [14], high-performance liquid chromatographic methods (HPLC) [15], high-performance liquid chromatography–mass spectrometry (HPLC–MS) [16], high-performance liquid chromatography–tandem mass spectrometry (HPLC–MS/MS) [17], ultra-performance liquid chromatography–tandem mass spectrometry (UPLC–MS/MS) [11,18], are routine methods for the determination of QNs, which have the advantages of superior sensitivity, selectivity and rapid analysis [19]. However, sample pretreatment methods prior to chromatographic analysis are essential due to the complexity of water samples and the trace concentration level of QNs. These methods include solid-phase extraction (SPE) [11,18], dispersive solid-phase extraction (DSPE) [20], magnetic solid-phase extraction (MSPE) [15], solid-phase microextraction (SPME) [21], dispersive liquid-liquid microextraction (DLLME) [22], and stirring bar adsorption extraction (SBSE) [23]. Among them, SPE is the most widely used because of its high enrichment factor. However, traditional SPE involves multiple steps of manual operation, which takes a long time, increasing the potential for human error and erratic recoveries.

Recently, automatic online SPE-chiral LC–MS/MS has been developed for the rapid determination of QNs in water using less sample volume (700 µL) and analysis time (14 min) [24], but its sensitivity needs to be further improved. Disk-based SPE, which has the advantages of high sample flow rates (at 50–100 mL/min), low risk of plugging, and big analyte mass transfer, is now used as a modified version of SPE in many application fields [25,26]. Due to current trends in legislation and normalization in the context of implementation of the Water Framework Directive, this method has been widely used in water analysis for various pollutants (e.g., heavy metal speciation, organic chlorinated pesticides (OCPs), neonicotinoid insecticides (NEOs), polycyclic aromatic hydrocarbons (PAHs), and polybrominated diphenyl ethers (PBDEs)) [25,26,27]. Hence, disk-based SPE has a promising role in the extraction and enrichment of trace QNs in environmental water. Peixoto et al. [28] proposed a down-scaled disk-based SPE system in which the eluate can be first screened by miniaturized fluorimetric reading, followed by individual determination of three QNs by LC–MS/MS, with a limit of detection (LOD) of 1 μg/L. Nevertheless, when compared to traditional SPE [11,18], the above miniaturized disk-based SPE procedure presents a higher LOD, and the concentrations of the three target ONs were all less than the LOD for estuarine waters from the Douro River [28]. More studies dedicated to the extraction and enrichment of various QNs in drinking water, river water, and seawater via disk-based SPE are needed.

The goal of this study is to develop a robust automated disk-based SPE coupled with stable isotope dilution UPLC–MS/MS for the rapid determination of 15 QNs in a large volume of drinking and environmental water samples. After optimization (e.g., adsorbent, ionic strength, Na_2_EDTA addition, sample pH) and validation, the proposed method was applied to detect QNs in various real water samples, including tap water, river water from Zhoushan, and seawater from Daiquyang and Yueqing Bay, East China. Then, the occurrence and ecological risks in the surveyed areas were also discussed and assessed, providing scientific methods and basis for pollution control and risk management of quinolone antibiotics.

## 2. Results and Discussion

### 2.1. Optimization of Automated Disk-Based Solid-Phase Extraction Procedures

#### 2.1.1. Selection of Disk Adsorbents

**Figure 1:**
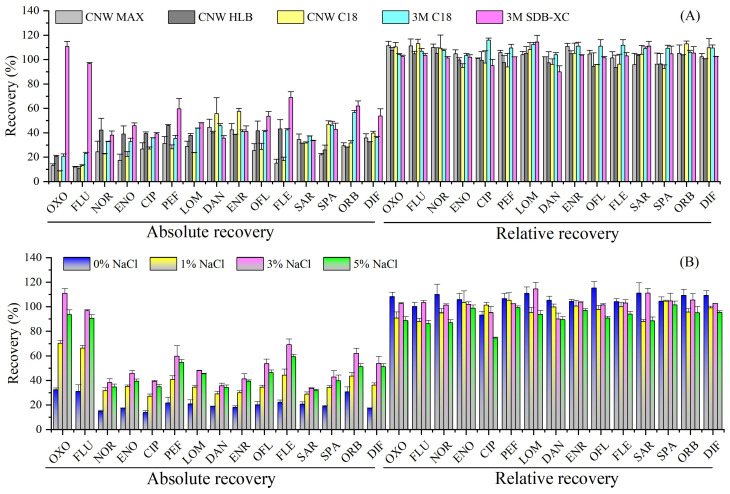
(**A**) Effect of disk adsorbents (n = 3); (**B**) effect of ionic strength (n = 3).

The selection of disk adsorbents plays a key role in improving both the enrichment and purification efficiency of target compounds in a complex matrix [26,27]. Five commercial SPE disks, including CNW MAX (mixed-mode cation exchange), CNW HLB (hydrophilic-lipophilic-balanced reverse phase polymer), CNW C18 (octadecyl), 3M C18 and 3M SDB-XC (polystyrenedivinylbenzene), were evaluated for the extraction of ultrapure water spiked with 20 ng/L of QNs (pH = 3, 3% NaCl) by the external standard method (absolute recovery) and the internal standard method (relative recovery). As shown in Figure 1A, the 3M SDB-XC disk had the highest absolute recoveries for the 15 QNs (range: 33–111%, mean: 55%), followed by 3M C18 (21–57%, 38%), CNW HLB (11–46%, 34%), CNW C18 (9–58%, 30%), and CNW MAX (12–45%, 27%), and the extraction efficiencies of 3M SDB-XC for OXO and FLU were significantly higher than those of the other four disks. It is worth noting that all five test disks obtained satisfactory relative recoveries (90–116%) after using internal standard correction, which may be attributed to the same molecular structures between the target compounds and their isotope-labeled internal standards (ILISs), as they exhibit the same adsorption properties during extraction and purification [29]. Therefore, the 3M SDB-XC disk was chosen in this study.

#### 2.1.2. Effect of Ionic Strength

The salinity of natural water varies greatly. Spiked ultrapure water samples (20 ng/L, pH = 3) with different amounts of NaCl (0–5%, *m*/*v*) were used to evaluate the influence of salinity on extraction efficiency. As seen in Figure 1B, the absolute recoveries significantly increased with NaCl concentration from 0% (range: 14–32%, mean: 21%) to 3% (33–111%, 55%), but slightly decreased at 5% (32–94%, 50%). However, there was no significant difference in the relative recoveries (75–115%) under different ionic strengths, which met the requirements of the analytical methods. Considering that the salinity of natural fresh water is extremely low and negligible, and the salinity of natural seawater in the open ocean usually ranges between 33 and 37‰ (mean: 35‰) [30], 3% NaCl should be added to fresh water, while no NaCl should be added to seawater.

#### 2.1.3. Effect of Na_2_EDTA and Sample pH

As shown in Supplementary Materials, Figure S1, the molecular structure of QNs usually contains a carboxylic group, a fluorine atom, and a piperazinyl or piperazine-derived group, which enhances their water solubility and ability to form stable complexes with metal ions, such as magnesium, calcium, aluminum, iron, and zinc [31]. During the extraction process, these formed complexes may affect the recoveries. Most of the previous literature improved the extraction efficiency of QNs by adding a certain amount of Na_2_EDTA to competitively form complexes with metal ions [11,32]. In this study, the absolute recoveries of the 15 QNs were significantly higher (76–194%, 126%) with 0.5 g/L Na_2_EDTA in spiked ultrapure water samples (20 ng/L, pH = 3, 3% NaCl) than those of the same spiked ultrapure but without Na_2_EDTA (33–111%, 55%). In this study, 0.5 g/L of Na_2_EDTA was added before water sample extraction.

As discussed above and based on the physicochemical properties of the QNs listed in Supplementary Materials, Table S1, the target 15 QNs had both carboxylic and piperazine groups, and can be divided into two groups according to their amphoteric properties: acid (pK_a_ 6.0–6.9) and piperazine (pK_a1_ 5.5–6.3, pK_a2_ 7.6–8.5). Under acidic conditions, they are mainly in neutral or cationic form, which is crucial for their retention on SPE adsorbents [33]. In most previous work, the pH values of water samples are usually adjusted to 2.5–4, resulting in QNs being in cationic form [33]. Therefore, considering that the pH value of real water environment is mostly in the range of 6–9, the pH of the water sample was adjusted to 3.0 before extraction in this study.

### 2.2. Matrix Effect

**Figure 2:**
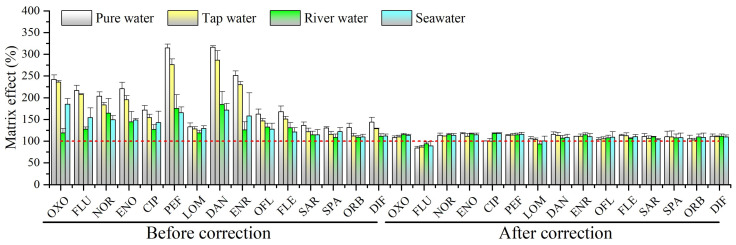
Matrix effects in 4 different water matrices.

UPLC–MS/MS is a powerful analytical technique due to its high sensitivity and selectivity, but it is susceptible to matrix effects (signal suppression/enhancement) [34]. In this study, the matrix effects (ME) were calculated using the formula ME (%) = (R_e_ − R_0_)/R_s_ × 100%, where R_e_, R_0_ and R_s_ were the signal intensity of spiked extracts, unspiked extracts and standard solution, respectively [29]. An ME value of 100% indicates no matrix effect, while values below 100% designate signal suppression, and values above 100% mean signal enhancement. As shown in Figure 2, signal enhancement was observed for the target 15 QNs, ranging from 108% to 316% in pure water, tap water, river water, and seawater. However, the matrix effects of the 15 QNs were minimized or eliminated by using the corresponding internal standard correction. It was demonstrated that the use of ILISs can effectively minimize/eliminate the matrix effect, without requiring any other processing, such as matrix calibration.

### 2.3. Evaluation of the Method Performance

**Figure 3:**
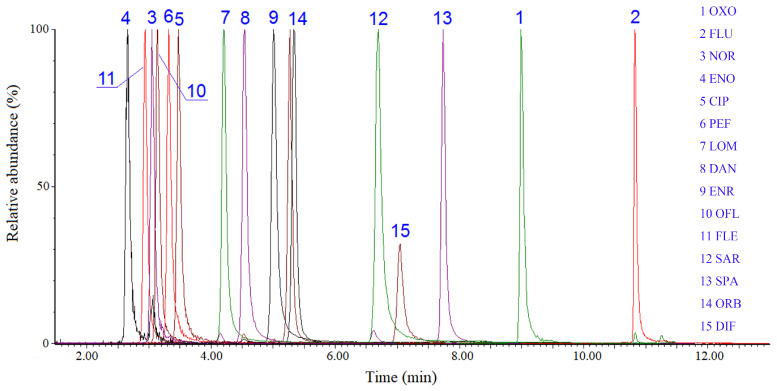
Chromatograms of the 15 QNs mixed standards by UPLC–MS/MS.

The developed automated disk-based SPE–UPLC–MS/MS method was validated in terms of linearity, limit of detection (LOD), limit of quantitation (LOQ), enrichment factors (EFs), and precision under optimum conditions [35] (Table 1). The calibration curves were constructed using each analyte’s concentration versus the peak area ratio of the target analyte to its corresponding ILIS. Wide linearity was achieved in the range of 0.05–100 μg/L for the 15 QNs, with correlation coefficients (r^2^) ranging from 0.9992 to 0.9999. The LODs and LOQs, defined as the signal-to-noise ratio of three and ten times, were in the ranges of 0.008–0.055 ng/L (S/N = 3) and 0.025–0.16 ng/L (S/N = 10), respectively. EFs were defined as the ratio of the analytes’ concentrations in the initial mobile phase after automated enrichment to the initial concentration of analytes in the water sample, ranging from 897 to 1136 for the target QNs. Intra- (n = 6) and inter-day (n = 6) precisions were calculated by extracting the analytes from ultrapure water samples at a concentration level of 20 ng/L, and relative standard deviations (RSDs) in the range of 2.7–10.8% and 4.6–12.3% were obtained, respectively (Table 1). A typical chromatogram of the 15 QNs standard mixture solution is shown in Figure 3. These results demonstrate that the present method exhibits high sensitivity and excellent repeatability.

Spiked recovery experiments were further conducted on the real water samples at three different concentration levels (1, 20, and 100 ng/L) to verify the accuracy of this method. As listed in Supplementary Materials, Table S2, the recoveries of the target QNs in tap water, river water, and seawater were in the ranges of 89–112%, 81–114%, and 82–112%, respectively, with RSDs of 1.5–12.1%, 0.2–13.3%, and 1.9–7.8% (n = 6), respectively, proving the good accuracy and precision of the proposed method.

Compared with the previous work (Table S3), the proposed automated disk-based SPE–UPLC–MS/MS method demonstrated comparable accuracy and precision, and provided similar LODs to manual SPE–UPLC–MS/MS (LODs: 0.150–0.256 ng/L) [36], DSPE–HPLC–MS/MS (0.02–0.06 ng/L) [20], and SPME–HPLC (0.14–0.61 ng/L) [21], but superior to those obtained using MSPE–HPLC (0.06–2.0 μg/L) [15], DLLME–HPLC (0.63–1.2 ng/L) [22], SBSE–HPLC (0.37–0.56 μg/L) [23], and online UPLC–MS/MS (1.77–14.4 ng/L) [37]. In addition to exhibiting high sensitivity, the developed method is also an effective, simple, and automated alternative for processing large-volume water samples in the field with satisfactory recovery. To conclude, the developed method is reliable and practical for the determination of QNs in real water samples.

### 2.4. Real Water Analysis

**Figure 4:**
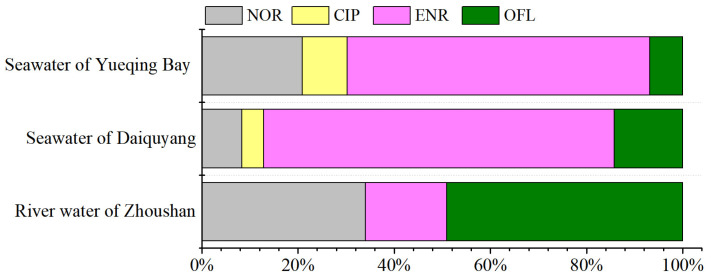
Composition characteristics of the detected QNs in river water of Zhoushan and seawater of Daiquyang and Yueqing Bay.

The proposed automated disk-based SPE–UPLC–MS/MS method was applied for the quantification of QNs in tap water, river water from Zhoushan, and seawater from Daiquyang and Yueqing Bay, East China. The results of the target QNs contents are listed in Supplementary Materials, Table S4, and Figure 4. None of the QNs were detected in tap water samples. Three QNs (NOR, ENR, and OFL) were detected in the river water from Zhoushan, with detection frequencies of 83–100%, and the total concentration of QNs (∑QNs) was in the range of 1.600–8.511 ng/L (mean: 4.426 ng/L). OFL was the predominant QN (mean: 2.171 ng/L, accounting for 49% of ΣQNs), followed by NOR (1.507 ng/L, 34%) and ENR (0.748 ng/L, 17%). Four QNs (NOR, CIP, ENR, and OFL) were detected in seawater, with detection frequencies of 100%. The ∑QNs concentrations in the seawater from Daiquyang (range: 2.183–16.421 ng/L, mean: 5.183) were slightly higher than those from Yueqing Bay (1.651–2.497 ng/L, 2.088 ng/L). In terms of composition, the first principal component was mainly ENR in both Daiquyang and Yueqing Bay, accounting for 63–73% of ∑QNs. The second principal component differed between samples: it was OFL (14%) in Daiquyang and NOR (21%) in Yueqing Bay. OFL, NOR, and CIP are the most used quinolones in hospitals and for livestock, while ENR is the most used drug in veterinary medicine [38]. The typical sample chromatograms of the detected QNs in Daiquyang seawater are shown in Figure S2.

In this study, the river water from Zhoushan was dominated by OFL, which may be related to the discharge of domestic sewage and medical wastewater from surrounding residents. The seawater from Daiquyang and Yueqing Bay were both dominated by ENR, which may be due to the development of marine aquaculture around the investigated sea area. ENR, CIP and NOR are commonly used antibiotics in aquaculture, especially ENR, which is the most widely used [39]. These results were consistent with other similar studies where OFL was dominant in the groundwater of the Hutuo River, China [40], influents and effluents of wastewater treatment plants (WWTPs) from Dalian, China [41], and influent, effluent, and surface water samples from Hangzhou, China [42]. In contrast, ENR was dominant in natural waters adjacent to mariculture areas in the Laizhou Bay, Bohai Sea [43]. Nevertheless, affected by sampling time, region, and drug use habits, the surface water of Pudong New Area of Shanghai, China, was dominated by NOR and CIP [44], lake water collected from Baiyangdian Lake, China, was dominated by FLU [45], influents of WWTPs from Durban, South Africa, were dominated by CIP [46], and aquaculture waters and surrounding water bodies located in north of Portugal were dominated by NOR [47]. In terms of concentration levels, different types of water bodies in different regions also differ greatly. The concentrations of QNs in river water and seawater in this study were below the middle level. The total concentrations of the detected QNs (mean: 2.088–5.183 ng/L) were significantly lower than those in influents and effluents from Dalian (1059.9 ± 1030.5 ng/L) [41], influents from Durban (15770 ng/L) [46], influent (2029 ng/L), effluent (778 ng/L), and surface water samples (83.5 ng/L) from Hangzhou [42], surface water of Pudong New Area of Shanghai (64.63–151.99 ng/L) [44], lake water from Baiyangdian Lake (153.1–955.24 ng/L) [45], groundwater of the Hutuo River (77.52–153.93 ng/L) [40], natural waters adjacent to mariculture areas in the Laizhou Bay, Bohai Sea (44.29–325.18 ng/L) [43], and aquaculture waters and surrounding water bodies of Portugal (33.4 ng/L) [47]. The total concentrations of QNs were comparable to coastal waters in Korea (8.28 ng/L) [48], surface water of the Yangtze River (1.38 ng/L) [49], aquaculture water (2.95 ± 3.26 ng/L), Qin River water (3.09 ± 2.89 ng/L), and seawater (4.32 ± 0.49 ng/L) near the Maowei Sea [50]. More attention should be paid to the occurrence of QNs in environment water.

### 2.5. Ecological Risk Assessment

The existing data on the occurrence of QNs in river water and seawater are crucial for evaluating health, ecological, and economic consequences. In this study, the ecological risk assessment is based on the risk quotient (RQ), which is the ratio of the measured environmental concentration in the collected water samples to the predicted no-effect concentration (PNEC) on non-target organisms [51]. The PNEC values were calculated by dividing the acute or chronic toxicity data by an assessment factor (AF) of 1000 or 100, respectively [51]. Generally, RQ was classified into low risk (RQ < 0.1), medium risk (0.1 < RQ < 1), and high risk (RQ > 1). Meanwhile, the total risk quotient (ΣRQ) was calculated based on the addition of each detected QN’s RQ to assess the combined contamination risk [52].

**Figure 5:**
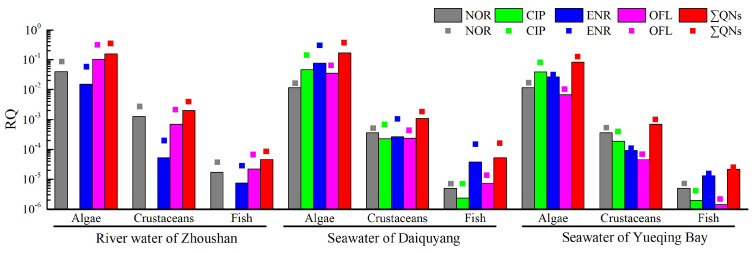
RQ values of aquatic organisms at different trophic levels.

**Figure 6:**
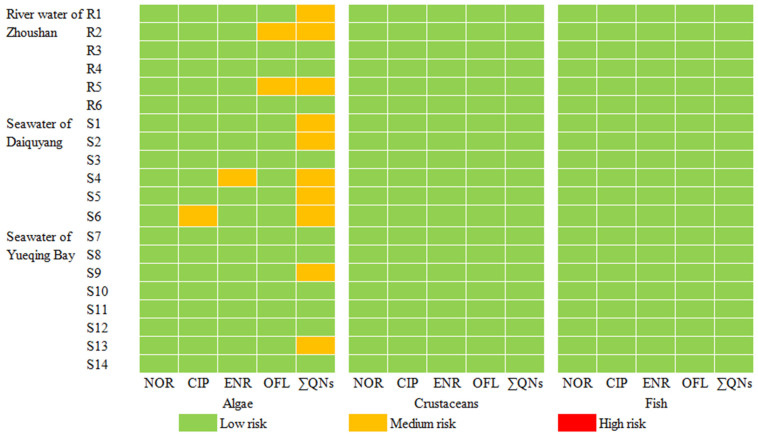
Ecological risks of the detected QNs to aquatic organisms at different trophic levels.

The details of the toxicity data and PNECs of the detected QNs are summarized in Supplementary Materials, Table S5, and the calculated RQ values for three different trophic levels of organisms (algae, crustaceans, and fish) are shown in Figure 5 and Figure 6. As can be seen, the maximum ΣRQ values of NOR, CIP, ENR and OFL for algae in the river water of Zhoushan and seawater of Daiquyang and Yueqing Bay are 0.36, 0.38, and 0.13, respectively, with average values of 0.16, 0.17, and 0.084, respectively. However, the maximum ΣRQ values for crustaceans and fish were both much less than 0.01, indicating that ΣQNs in the river water and seawater in the surveyed areas exhibited a low-to-medium risk to algae, and a low risk to crustaceans and fish. Overall, the ΣRQ values decreased continuously with the increase in trophic levels of aquatic organisms. The ecological risk of QNs to algae was mainly due to OFL in river water, while ENR and CIP were the main contributors in seawater, with maximum RQ values of 0.31, 0.31 and 0.14, respectively. More attention should be paid to the distribution level and exposure risk of these QNs in the environment. Antibiotic residues not only affect the behavior, reproductive capacity and growth of algae, crustaceans and fish, but also produce genotoxicity, even at trace levels, thus, antibiotic contamination control in environment water cannot be ignored [53]. In addition to the ecotoxicological effects, their long-term presence in the environment also creates selection pressure on microorganisms and contributes to the emergence of multi-drug resistant-bacteria [54].

## 3. Materials and Methods

### 3.1. Chemicals and Reagents

A total of 15 QNs standards (>99% purity), i.e., oxolinic acid (OXO), flumequine (FLU), norfloxacin (NOR), enoxacin (ENO), ciprofloxacin (CIP), pefloxacin (PEF), lomefloxacin (LOM), danofloxacin (DAN), enrofloxacin (ENR), ofloxacin (OFL), fleroxacin (FLE), sarafloxacin hydrochloride (SAR), sparfloxacin (SPA), orbifloxacin (ORB), and difloxacin (DIF) were purchased from ANPEL Laboratory Technologies (Shanghai, China). Their corresponding 15 ILISs, including OXO-D_5_, FLU-^13^C_3_, NOR-D_5_, ENO-D_8_, CIP-D_8_, PEF-D_5_, LOM-D_5_, DAN-D_3_, ENR-D_5_, OFL-D_3_, FLE-D_3_, SAR-D_8_, and DIF-D_3_, were obtained from Alta Scientific Co., Ltd. (Tianjin, China), and SPA-D_4_ and ORB-D_4_ were purchased from Toronto Research Chemicals (Toronto, ON, Canada). Methanol was used to prepare the stock standard solutions of the 15 QNs (1 mg/L) and 15 ILISs (1 mg/L). The standard solutions were stored in amber glass bottles at −20 °C. Calibration standard solutions were prepared daily by diluting the stock solutions with the initial mobile phase.

HPLC-grade methanol and formic acid were obtained from Merck (Darmstadt, Germany), and sodium chloride (NaCl), ammonium acetate, ethylenediaminetetraacetic acid disodium salt (Na_2_EDTA) were obtained from Sigma-Aldrich (St. Louis, MO, USA). Ultrapure water (18.2 MΩ/cm) was prepared by a Milli-Q Plus 185 system (Millipore Corporation, Burlington, MA, USA). 3M C18 (47 mm) and 3M SDB-XC (47 mm) disks from 3M Empore (St. Paul, MN, USA), and CNW C18 (1 g), CNW MAX (1 g), CNW HLB (1 g) disks from CNW Technologies (Duesseldorf, Germany) were used for disk-based SPE extraction.

### 3.2. Sampling and Preparation

A total of twenty-six water samples were collected in May 2023. Six tap water samples were collected from local families. Six river water samples were collected from the urban river in Zhoushan (R1–R6). Fourteen seawater samples were collected from Daiquyang (S1–S6) and Yueqing Bay (S7–S14), East China. The sampling locations are shown in Figure S3. The collected river water or seawater samples were filtered through 0.45 μm membranes to remove suspended solid particles and then stored at 4 °C until automated disk-based SPE extraction.

### 3.3. Automated Disk-Based Solid-Phase Extraction

Sample extraction was carried out using an automated cartridge-disk universal SPE system (LabTech, Shanghai, China) (Supplementary Materials, Figure S4), which can process six samples simultaneously within 60 min. In brief, the disks were conditioned by adding 10 mL of methanol and 10 mL of acidified ultrapure water (pH 3.0). Then, 1.0 L of the filtered water sample with 0.5 g Na_2_EDTA and 20 ng of the 15 ILISs (pH = 3) was passed through the preconditioned disk at a flow rate of 50–100 mL/min, followed by 15 mL of ultrapure water. After sample loading and rinsing, the SPE disks were dried under N_2_-blowdown for 15 min and eluted with 10 mL of methanol. Finally, the collected eluents were evaporated to dryness with a stream of nitrogen at 50 °C, reconstituted with 1 mL of the initial mobile phase, and filtered through 0.22 μm filter for UPLC–MS/MS analysis.

### 3.4. Instrumental Analysis

Chromatographic analysis was performed using a Waters Acquity UPLC I-Class system (Waters, Milford, MA, USA) coupled with a Xevo TQ-S triple quadrupole mass spectrometer (Waters, Manchester, UK) in multiple reaction monitoring (MRM) modes following our previous work [29,55]. In detail, chromatographic separation was carried out in a Waters BEH C18 column (2.1 mm × 100 mm, 1.7 μm), thermostated at 40 °C using a mobile phase of 2 mM ammonium acetate solution containing 0.1% formic acid (eluent A) and acetonitrile (eluent B). The flow rate was 0.30 mL/min, and the injection volume was 5 μL, with a gradient elution as follows: 10% B (0−1.5 min), 10–12.5% B (1.5−6.5 min), 12.5–30% B (6.5−9.5 min), 30–40% B (9.5−10.5 min), 40–90% B (10.5−10.7 min), 90% B (10.7−11.5 min), 90–10% B (11.5−11.8 min), and finally 10% B (11.8−15 min).

The MS/MS was operated in positive electrospray ionization (ESI+), with operating conditions as follows: capillary voltage at 3.0 kV; desolvation temperature at 600 °C; source temperature at 150 °C; desolvation gas flow at 800 L/h; and cone gas flow at 150 L/h. Nitrogen (99.99%) was used as the desolvation and cone gas, and argon (99.9999%) as collision gas. Table 2 shows the MRM transition parameters for each compound.

## 4. Conclusions

In summary, an automated disk-based SPE–UPLC–MS/MS method was developed for the simultaneous enrichment and quantification of 15 QNs within 60 min/6 samples using a 3M SDB-XC disk. Compared with SPE cartridges, the disk SPE procedure is faster and time-saving, making it highly suitable for the extraction of large-volume water samples both in the laboratory and in the field. The proposed method was validated in terms of calibration linearity, method detection limit, recovery, and precision. The results demonstrated that this method is effective and sensitive for the determination of QNs in drinking and environmental waters. None of the QNs were detected in tap water samples. Three and four QNs were detected in river water and seawater, respectively. OFL was the dominant QN detected in the river water of Zhoushan, while ENR was the main component in the seawater of Daiquyang and Yueqing Bay. Risk assessment revealed that ΣQNs posed a low risk to crustaceans and fish, but a low-to-medium risk to algae. Specifically, OFL was the main risk contributor in river water, while ENR and CIP were the main contributors in seawater. Therefore, the antibiotic pollution in environmental water cannot be ignored, and long-term monitoring of antibiotic residues is required. Considering the potential accumulation of quinolone antibiotics by aquatic organisms from water, further studies are also needed to assess human exposure to antibiotics via aquatic food. For instance, attention should be paid to the occurrence, bioaccumulative ability, estimated daily human intake, and dietary assessments of different fish tissues for antibiotics at actual environmental concentrations. Moreover, the eco-toxicity of antibiotics and their resistance in aquatic ecosystem need to be investigated.

## Figures and Tables

**Table 1:** Analytical characteristics of the proposed method.

Analyte	ILIS	Linear Range (μg/L)	Calibration Curve	r^2^	LOD ^a^ (ng/L)	LOQ ^b^ (ng/L)	EFs	Precision, RSD (%, n = 6)	
								Intra-Day	Inter-Day
OXO	OXO-D_5_	0.05−100	y = 0.75x − 0.008	0.9998	0.014	0.045	1022	4.4	8.7
FLU	FLU-^13^C_3_	0.05−100	y = 1.10x − 0.001	0.9998	0.012	0.040	1032	7.7	12.3
NOR	NOR-D_5_	0.05−100	y = 1.14x − 0.012	0.9999	0.026	0.080	1009	7.9	9.8
ENO	ENO-D_8_	0.05−100	y = 1.24x − 0.052	0.9992	0.055	0.160	1012	10.8	11.6
CIP	CIP-D_8_	0.05−100	y = 1.01x + 0.025	0.9997	0.025	0.080	950	4.1	4.6
PEF	PEF-D_5_	0.05−100	y = 1.17x + 0.163	0.9996	0.018	0.055	1018	8.6	9.9
LOM	LOM-D_5_	0.05−100	y = 1.18x + 0.135	0.9992	0.022	0.065	1136	7.7	11.1
DAN	DAN-D_3_	0.05−100	y = 1.08x + 0.053	0.9999	0.008	0.025	897	6.9	9.8
ENR	ENR-D_5_	0.05−100	y = 1.13x + 0.011	0.9994	0.024	0.075	1031	3.9	8.2
OFL	OFL-D_3_	0.05−100	y = 1.14x + 0.075	0.9999	0.022	0.070	1012	2.9	7.7
FLE	FLE-D_3_	0.05−100	y = 1.19x + 0.008	0.9997	0.022	0.070	1028	2.7	7.8
SAR	SAR-D_8_	0.05−100	y = 1.21x + 0.016	0.9996	0.022	0.070	1108	4.9	9.7
SPA	SPA-D_4_	0.05−100	y = 1.06x − 0.006	0.9992	0.02	0.060	1039	4.4	8.1
ORB	ORB-D_4_	0.05−100	y = 0.59x + 0.001	0.9997	0.02	0.060	1048	4.0	7.6
DIF	DIF-D_3_	0.05−100	y = 0.79x − 0.008	0.9996	0.024	0.075	1023	5.0	8.3

^a^ LOD (S/N = 3); ^b^ LOQ (S/N = 10).

**Table 2:** The MS/MS conditions of the 15 QNs.

Analyte	Retention Time (min)	Precursor Ion (*m*/*z*)	Product Ion (*m*/*z*)	Cone Voltage (V)	Collision Energy (eV)
OXO	9.01	262	216, 244 *	23	30, 20
FLU	10.83	262.1	202, 244 *	29	32, 18
NOR	3.06	320.1	233, 276.1 *	30	25, 18
ENO	2.65	321.1	232, 303.1 *	32	30, 35
CIP	3.48	332.2	288.2, 314.2 *	28	16, 15
PEF	3.32	334.1	290.1, 316.1 *	34	15, 20
LOM	4.20	352.1	265.1 *, 308.1	31	22, 15
DAN	4.53	358.2	96, 340.2 *	34	25, 22
ENR	5.01	360.4	245.2, 316.2 *	32	18, 20
OFL	3.14	362.1	261.1, 318.1 *	30	20, 20
FLE	2.94	370.1	269.1, 326.1 *	34	25, 19
SAR	6.69	386.2	299.1, 342.1 *	33	27, 18
SPA	7.74	393.2	292.1 *, 349.1	37	24, 20
ORB	5.33	396.1	295.1 *, 352	36	22, 15
DIF	7.05	400.2	299, 356.1 *	37	27, 21

* quantitative ion.

## Data Availability

Data are contained within the article and Supplementary Materials.

## Supplementary Materials

The following supporting information can be downloaded at: https://www.mdpi.com/article/10.3390/molecules29194611/s1, Figure S1: Chemical structures of the 15 QNs; Figure S2. Typical chromatograms of detected QNs in Daiquyang seawater by UPLC-MS/MS; Figure S3. Map of sampling locations for river water (R1−R6) and seawater (S1–S14), East China; Figure S4. Automatic cartridge-disk universal solid phase extraction system (LabTech, China). Table S1: The physicochemical properties of 15 QNs.; Table S2. Recoveries of 15 target QNs at three spiking levels in tap water, river water, and seawater by the proposed automated disk-based SPE UPLC-MS/MS method; Table S3. The comparison of the proposed method with other methods for QNs detection in water samples; Table S4. Concentrations (ng/L) of detected QNs in river water of Zhoushan and seawater of Daiquyang and Yueqing Bay (ng/L); Table S5. Toxicity data and PNECs of detected QNs on three different trophic levels aquatic organisms. References [56,57,58,59,60,61,62,63,64] are cited in the supplementary materials.
