# Supplementary material for: Determination and Ecological Risk Assessment of Quinolone Antibiotics in Drinking and Environmental Waters Using Fully Automated Disk-Based SPE Coupled with UPLC–MS/MS
Source: Molecules. 2024 Sep 28;29(19):4611. doi: 10.3390/molecules29194611 (PMC11477713; doi:10.3390/molecules29194611)
Supplement: Supplementary file 1 [file molecules-29-04611-s001.zip › molecules-3219598-supplementary.pdf]

SUPPORTING INFORMATION

Determination and Ecological Risk Assessment of Quinolone Antibiotics in Drinking and Environmental Waters Using Fully Automated Disk-Based SPE Coupled with UPLC–MS/MS

Hongmei Hu [1], Xingyu Da [1], Zhenhua Li [1,*], Tiejun Li [1], Xiaoning Zhang [2,*], Tianbin Bian [3], Yanjian Jin [4], Kaida Xu [1] and Yuanming Guo [1]

1 Key Laboratory of Sustainable Utilization of Technology Research for Fisheries Resources of Zhejiang Province, Zhejiang Marine Fisheries Research Institute, Zhoushan 316021, China; huhm@zju.edu.cn (H.H.);

2 State Key Laboratory of Resource Insects, College of Sericulture, Textile and Biomass Sciences, Southwest University, Chongqing 400715, China

3 Hangzhou Center for Disease Control and Prevention, Hangzhou 310021, China

4 Zhejiang Marine Ecology and Environment Monitoring Center, Zhoushan 316021, China

* Correspondence: lzh0580@zjou.edu.cn (Z.L.); xzhang@swu.edu.cn (X.Z.); Tel.: +86-58-0229-9833 (Z.L.); +86-023-6825-1309 (X.Z.); Fax: +86-58-0229-9881 (Z.L.); +86-023-6825-0191 (X.Z.)

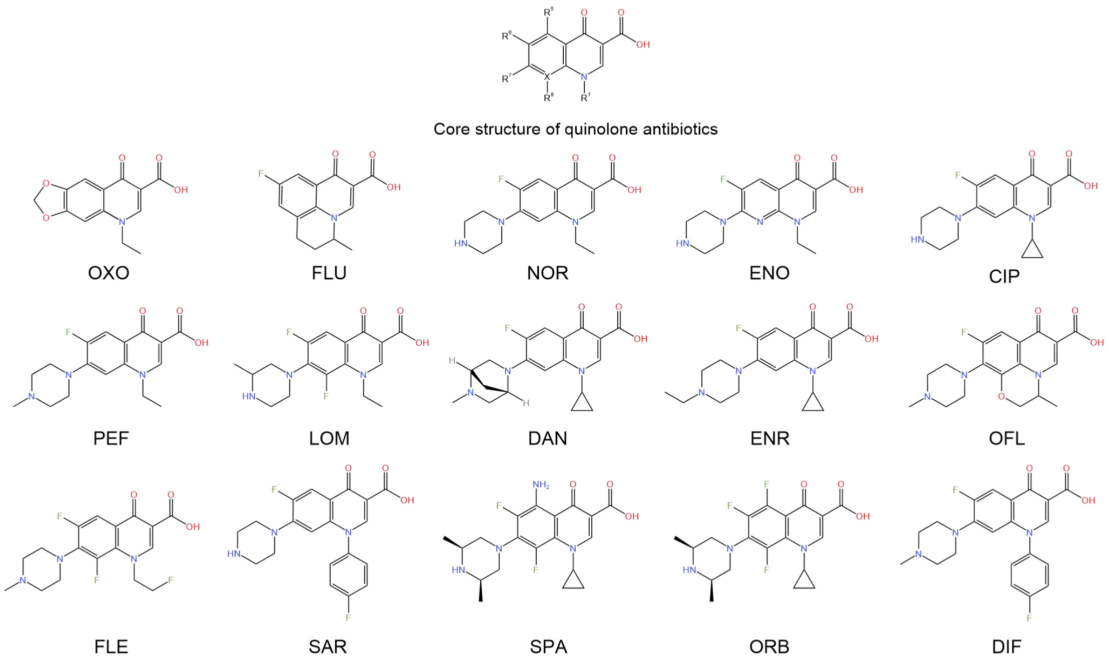

Figure S1. Chemical structures of the 15 QNs.

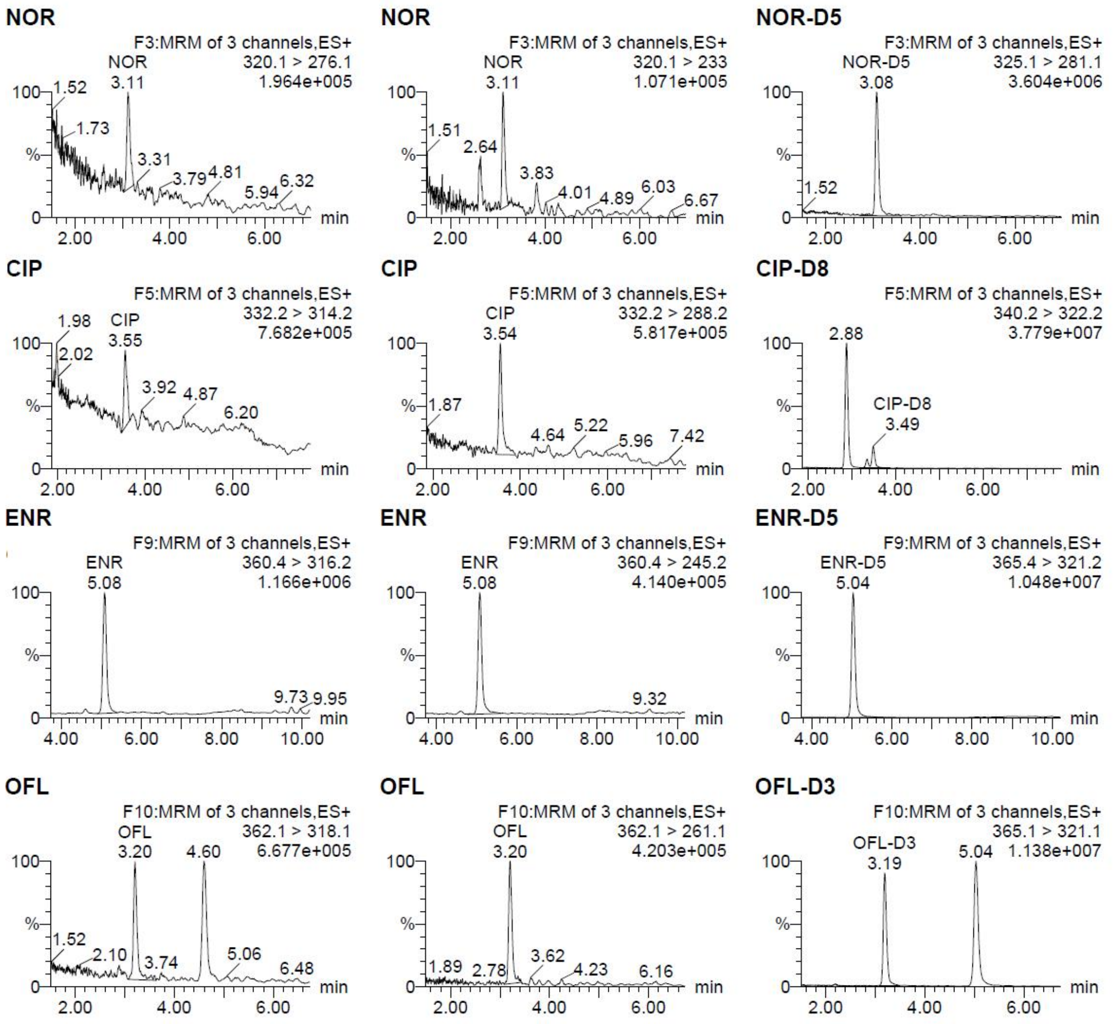

Figure S2. Typical chromatograms of detected QNs in Daiquyang seawater by UPLC-MS/MS.

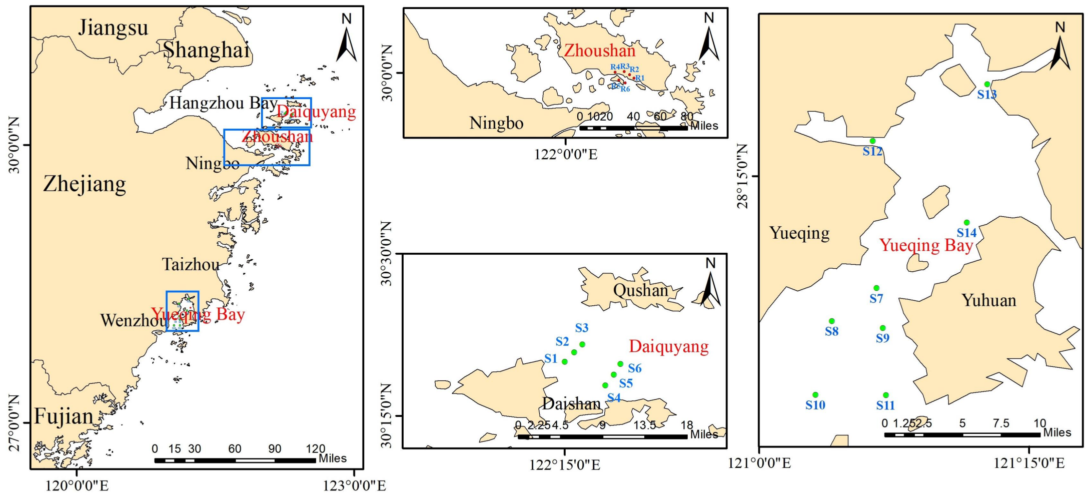

Figure S3. Map of sampling locations for river water (R1−R6) and seawater (S1−S14), East China.

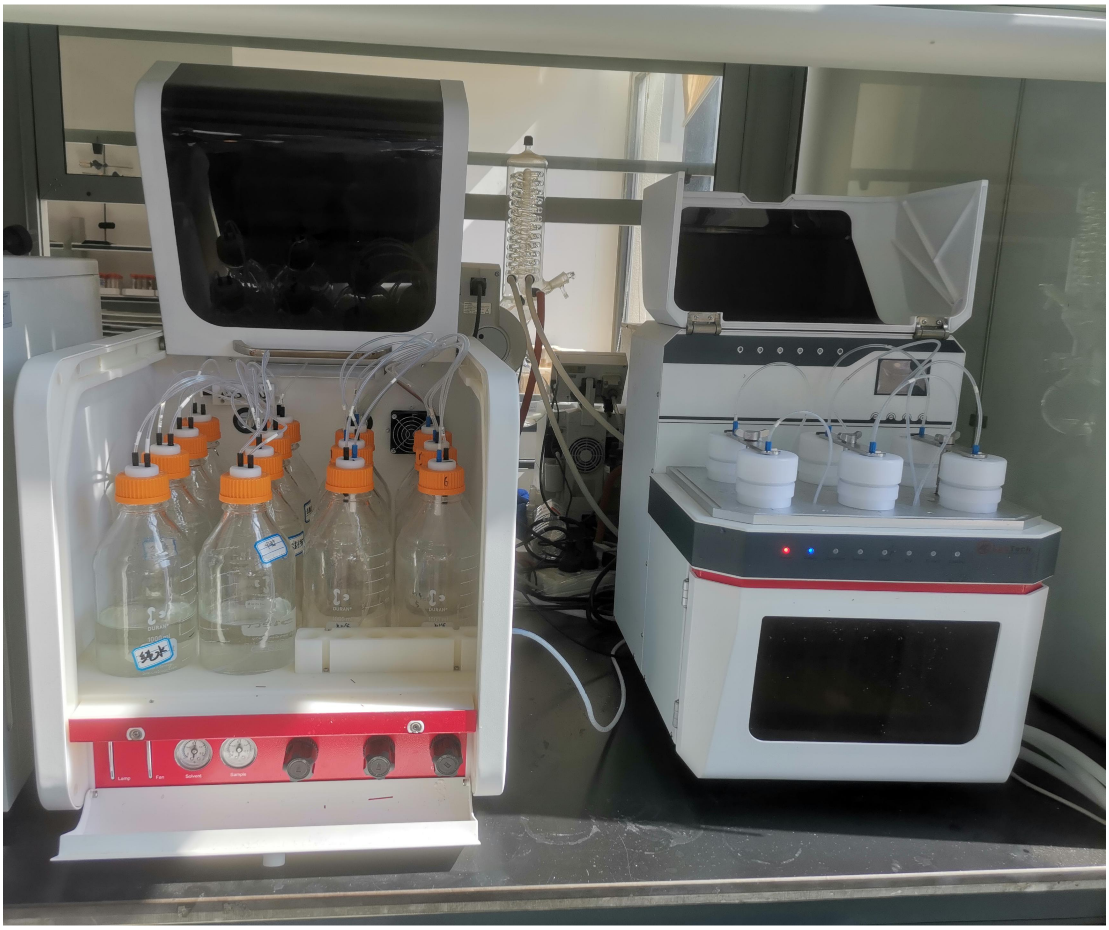

Figure S4. Automatic cartridge-disk universal solid phase extraction system (LabTech, China).

Table S1. The physicochemical properties of 15 QNs.

Abbreviation	Full Name	CAS No.	Chemical formula	Molecular weight	Log K_{OW}	pKa
OXO	oxolinic acid	14698-29-4	$C_{13}H_{11}NO_5$	261.23	0.94	6.90
FLU	flumequine	42835-25-6	$C_{14}H_{12}FNO_3$	261.25	1.60	6.50
NOR	norfloxacin	70458-96-7	$C_{16}H_{18}FN_3O_3$	319.33	-1.03	3.11/6.10/8.60/10.56
ENO	enoxacin	74011-58-8	$C_{15}H_{17}FN_4O_3$	320.32	-0.20	5.50/8.59
CIP	ciprofloxacin	85721-33-1	$C_{17}H_{18}FN_3O_3$	331.34	0.28	3.10/6.14/8.70/10.58
PEF	pefloxacin	70458-92-3	$C_{17}H_{20}FN_3O_3$	333.36	0.27	5.66/6.47
LOM	lomefloxacin	98079-51-7	$C_{17}H_{19}F_2N_3O_3$	351.35	-0.30	5.00/5.87/9.23
DAN	danofloxacin	112398-08-0	$C_{19}H_{20}FN_3O_3$	357.38	0.44	6.07/8.56
ENR	enrofloxacin	93106-60-6	$C_{19}H_{22}FN_3O_3$	359.39	0.70	3.86/6.19/7.59/9.86
OFL	ofloxacin	82419-36-1	$C_{18}H_{20}FN_3O_4$	361.37	-0.39	5.97/8.28
FLE	fleroxacin	79660-72-3	$C_{17}H_{18}F_3N_3O_3$	369.34	0.24	5.44/6.06
SAR	sarafloxacin	98105-99-8	$C_{20}H_{17}F_2N_3O_3$	385.36	1.07	5.60/8.20
SPA	sparfloxacin	110871-86-8	$C_{19}H_{22}F_2N_4O_3$	392.40	2.50	5.75/8.79
ORB	orbifloxacin	113617-63-3	$C_{19}H_{20}F_3N_3O_3$	395.38	2.37	NA
DIF	difloxacin	98106-17-3	$C_{21}H_{19}F_2N_3O_3$	399.39	0.89	5.66/7.24

Table S2. Recoveries of 15 target QNs at three spiking levels in tap water, river water, and seawater by the proposed automated disk-based SPE UPLC-MS/MS method.

Analyte	Tap water (n=6)						River water (n=6)						Seawater (n=6)					
	Spiked: 1 ng/L		Spiked: 20 ng/L		Spiked:100 ng/L		Spiked: 1 ng/L		Spiked: 20 ng/L		Spiked: 100 ng/L		Spiked: 1 ng/L		Spiked: 20 ng/L		Spiked: 100 ng/L	
	Recovery (%)	RSD (%)	Recovery (%)	RSD (%)	Recovery (%)	RSD (%)	Recovery (%)	RSD (%)	Recovery (%)	RSD (%)	Recovery (%)	RSD (%)	Recovery (%)	RSD (%)	Recovery (%)	RSD (%)	Recovery (%)	RSD (%)
OXO	112	6.3	99	7.8	89	4.9	106	5.5	103	2.8	93	1.2	82	3.2	88	2.5	95	2.3
FLU	105	7.4	97	11.1	92	6.8	108	8	112	8	93	1.3	95	4.2	92	3.6	101	1.9
NOR	101	9.3	97	7.3	93	8.2	105	8.6	94	5.2	110	7.5	93	6.4	90	4.1	106	5.8
ENO	106	10.5	96	9	102	7.9	100	13.3	113	4.8	97	9.3	92	5.9	96	2.5	98	6.5
CIP	98	6.3	95	1.5	96	2.6	95	4.3	89	1.6	85	12.6	99	4.7	98	4.2	89	7.8
PEF	102	8.4	98	9.3	90	4.8	95	9.4	110	4.2	95	4	96	2.8	92	2.3	93	3.8
LOM	95	6.9	97	9.5	93	6.4	107	9.5	104	0.4	91	2.4	98	3.9	98	1.9	95	4.2
DAN	97	12.1	93	8.8	96	6.9	101	7.4	102	0.2	89	5	112	4.7	96	2.1	92	3.7
ENR	105	4.8	97	9.8	98	8.9	102	3.8	111	2.7	88	3.4	102	6.3	89	5.6	93	4.3
OFL	94	3.5	94	8.4	92	7.5	99	2.8	111	5.6	90	3.6	96	4.6	92	3.2	94	3.9
FLE	101	4.6	93	8.4	93	6.9	98	2.8	114	6	91	5.3	92	5.5	95	4.1	93	3.4
SAR	102	6.2	97	9.9	94	4.2	105	5	107	5.5	81	3.3	90	3.7	88	3.3	93	2.8
SPA	98	9.6	92	10.9	90	7.6	100	3	106	1.2	99	5.8	97	3.2	93	1.9	95	3.4
ORB	105	6.8	99	8.2	93	6.2	103	4.1	101	1.3	98	0.6	106	3.4	91	2.8	94	2.7
DIF	102	9.4	98	7.8	87	2.7	105	4.7	112	7.4	88	2.2	101	4.7	98	3.4	93	3

Table S3. The comparison of the proposed method with other methods for QNs detection in water samples.

Method	N*	Sample volume	Sample pretreatment	sorbent	Extract solvent	Elution or disperser solvent	Processing time (min)	LOD	Recovery (%)	RSD (%)	Ref.
MSPE-HPLC	5	50 mL	/	15 mg of $MnFe_2O_4@TiO_2$	/	5 mL of methanol/ammonia solution (v/v= 80/20)	~20 min	0.6–2.0 μg/L	78.9–105.8	<9	[15]
DSPE-HPLC-MS/MS	4	30 mL	/	50 mg of COFs-aerogel	/	methanol	~30 min	0.02–0.06 ng/L	81.4–96.2	<9	[20]
SPME-HPLC	5	16 mL	pH adjustment 4.0	MOF-monolithcomposite-based capillary microextraction column (MM-CMC)	/	50 μL desorption solvent (methanol/aqueous solution of 2.0%TFA=90/10 (v/v))	~45 min	0.14–0.61 ng/L	80.1–120	<10	[21]
DLLME-HPLC	3	5 mL	pH adjustment 8.5	/	750 μL of Tricaprylymethylammonium chloride (Aliquat 336)	1170 μL of methanol	~18 min	0.63–1.2 ng/L	99.1–108	<6	[22]
SBSE-HPLC	4	100 mL	pH adjustment 5.0	poly (methacrylic acid-3-sulfopropyl ester potassium saltdivinylbenzene) monolithic material (MADB)	/	methanol/water (pH value 1.3) (v/v = 80/20)	~100 min	0.37–0.56 μg/L	62.2–72.9	<11	[23]
manual SPE UPLC-MS/MS	4	1 L	Addition Na_2EDTA, pH adjustment 2.0	200 mg of Oasis HLB	/	10 mL of 2% formic acid solution in methanol/acetonitrile(4:1, v/v)	> 250 min	0.150–0.256	75.7–95.8	<10	[36]
On-line SPE UPLC-MS/MS	8	10 mL	Addition formic acid	HyperSep Retain PEP (porous polystyrene divinylbenzene)	/	methanol/acetonitrile(1:1, v/v)	20 min	1.77–14.4 ng/L	50–150	<22	[37]
automated disk-based SPE UPLC-MS/MS	15	1 L	Addition NaCl and Na_2EDTA, pH adjustment 3.0	3M SDB-XC (47 mm) disk	/	10 mL of methanol	~60 min	0.008–0.055	81–114	<14	Present work

* Number of QNs evaluated.

Table S4. Concentrations (ng/L) of detected QNs in river water of Zhoushan and seawater of Daiquyang and Yueqing Bay (ng/L).

		NOR	CIP	ENR	OFL	∑QNs
River water of Zhoushan (n=6)	Min	ND	ND	0.089	0.240	1.600
	Max	3.325	ND	2.908	6.562	8.511
	Mean	1.507	ND	0.748	2.171	4.426
	DF(%)	83	0	100	100	100
Seawater of Daiquyang (n=6)	Min	0.202	0.039	1.107	0.388	2.183
	Max	0.632	0.698	15.249	1.345	16.421
	Mean	0.434	0.235	3.776	0.738	5.183
	DF(%)	100	100	100	100	100
Seawater of Yueqing Bay (n=8)	Min	0.141	0.071	1.081	0.070	1.651
	Max	0.652	0.405	1.583	0.214	2.497
	Mean	0.437	0.194	1.315	0.142	2.088
	DF(%)	100	100	100	100	100

DF, detection frequency.
ND, not detected.

Table S5. Toxicity data and PNECs of detected QNs on three different trophic levels aquatic organisms.

Analytes	Class	Species	Toxicity data (mg/L)		PNEC (ng/L)	References
			EC_{50}/LC_{50}	NOEC		
NOR	Alage	*Microcystis wesenbergii*	0.038		38	[56]
	Crustaceans	*Daphnia magna*		0.12	1200	[57]
	Fish	*Carassius auratus*		8.78	87800	[58]
CIP	Alage	*Microcysti aeruginosa*	0.005		5	[59]
	Crustaceans	*Daphnia magna*	1.034		1034	[60]
	Fish	*Danio rerio*	100		100000	[61]
ENR	Alage	*Microcystis aeruginosa*	0.049		49	[62]
	Crustaceans	*Litopenaeus vannamei*	14.3		14300	[63]
	Fish	*Pimephales promelas*		10	100000	[62]
OFL	Alage	*Microcystis aeruginosa*	0.021		21	[62]
	Crustaceans	*Ceriodaphnia dubia*	3.13		3130	[64]
	Fish	*Pimephales promelas*		10	100000	[62]
